# Functional effect of adiponectin and body composition assessment in lung cancer subjects after video‐assisted thoracoscopic surgery (VATS) lobectomy

**DOI:** 10.1111/1759-7714.15260

**Published:** 2024-12-09

**Authors:** Gaetana Messina, Giovanni Natale, Alfonso Fiorelli, Maria Antonietta Puca, Fiorenzo Moscatelli, Vincenzo Monda, Marcellino Monda, Marina Di Domenico, Carminia Maria Della Corte, Gabriella Marsala, Giuseppe Vicario, Carmine Dalia, Paola Bassi, Beatrice Leonardi, Antonella De Maria, Antonietta Monda, Giovanni Messina, Antonietta Messina, Rita Polito

**Affiliations:** ^1^ Department of Translational Medicine Università degli Studi della Campania “Luigi Vanvitelli” Naples Italy; ^2^ Department of Human Sciences Telematic University Pegaso Naples Italy; ^3^ Department of Economics, Law, Cybersecurity, and Sports Sciences University of Naples “Parthenope” Naples Italy; ^4^ Department of Experimental Medicine Section of Human Physiology and Unit of Dietetics and Sports Medicine, University of Campania “Luigi Vanvitelli” Naples Italy; ^5^ U.O.C. of Conventional Pharmaceuticals Catania Italy; ^6^ Department of Experimental Medicine University of Camapania “Luigi Vanvitelli” Naples Italy; ^7^ Department of Human Sciences and Quality of Life Promotion Telematic University San Raffaele Rome Italy; ^8^ Department of Precision Medicine University of Campania “Luigi Vanvitelli” Naples Italy; ^9^ Department of Clinical and Experimental Medicine University of Foggia Foggia Italy

**Keywords:** body composition, inflammation, lung cancer, nutrition, video‐assisted thoracoscopic surgery (VATS) lobectomy, weight loss

## Abstract

**Background:**

Lung cancer is a pathology with an important incidence. It is a multifactorial disease characterized by epigenetic and nutritional factors. Indeed, there is a strong association between adipose tissue and the pulmonary system, and low‐grade inflammation of obese and/or overweight subjects have a pivotal role in lung cancer establishment.

**Methods:**

In this study, we analyzed body composition through bioelectrical impedance analysis (BIA) and biochemical parameters such as glycemic and lipidic profile, inflammation profile and adiponectin serum levels in 30 patients (19 male; 11 women) undergoing video‐assisted thoracoscopic surgery (VATS) lobectomy for lung cancer from September 2021 to May 2022 at the Thoracic Unit of Luigi Vanvitelli University of Naples. A control group were also recruited (15 male; 15 female) consisting of age and sex matched volunteered subjects at the Thoracic Unit of Luigi Vanvitelli University of Naples. The control group and lung cancer patients were monitored for anthropometric and biochemical parameters before VATS lobectomy. Furthermore, the lung cancer patients were also monitored after 6 months of surgery.

**Results:**

Body composition is modified after surgery and also albumin and C‐reactive protein (CRP) serum levels. In the overweight patients in our study, adiponectin levels were found to be reduced compared with the control group and increased in the same patients after VATS lobectomy.

**Conclusions:**

Tumor removal as well as weight loss could affect adiponectin levels, and thus also a reduction in inflammation. In addition, weight loss could also be due to a psychological condition given by the intervention and not to malnutrition related to therapy.

## Graphical abstract

Body composition and adipose tissue play a role in the predisposition and progression of lung cancer. Patients with lung carcinoma exhibited lower levels of adiponectin compared with the control population. Additionally, adiponectin levels in patients undergo changes after surgical resection of lung carcinoma. Disturbances in the adiponectin system in lung carcinoma may lead to the modulation of the antiproliferative and anti‐inflammatory effects induced by adiponectin, offering potential new therapeutic options for patients.

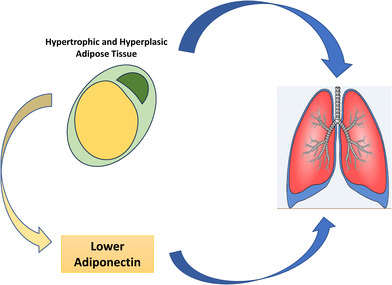

## INTRODUCTION

Lung cancer is a severe condition that remains a significant contributor to global cancer‐related deaths.^1^, ^2^ Additionally, it has a high mortality rate as it is often diagnosed at an advanced stage. The processes leading to this neoplasia are varied: genetic factors and lifestyle‐related factors such as tobacco smoking, alcohol consumption, diet, and physical activity increase and/or decrease the risk of developing lung cancer. This neoplastic disease is multifactorial, involving genetic, environmental, and nutritional factors. Recent research has shed light on the strong connection between the pulmonary epithelium and adipose tissue, where the secretion of adipokines, including adiponectin, is disrupted in many lung‐related pathological conditions.^2^ Adipose tissue, particularly in individuals overweight or obese, plays a critical role in promoting a state of chronic inflammation, and this inflammatory imbalance is also evident in the lung.^3^, ^4^, ^5^ Obesity arises from a combination of factors, including imbalanced energy intake, which affects metabolic parameters such as blood glucose levels, total cholesterol, and inflammatory markers.^1^ Consequently, this multifaceted disease is linked to other seemingly unrelated chronic conditions such as type 2 diabetes, cardiovascular diseases, and various cancers. Obese individuals commonly experience cancers of the esophagus, lungs, colon‐rectum, breast, endometrium, and kidney.^6^, ^7^ In obesity, both visceral and subcutaneous adipose tissues undergo alterations. The alteration of visceral adiposity closely correlates with the low‐grade chronic and systemic inflammation seen in obese individuals, increasing their susceptibility to various types of cancer, as previously reported.^5^, ^6^, ^7^ Due to the endocrine role of adipose tissue, which produces adipocytokines, it can regulate the functionality, energy metabolism, and inflammatory homeostasis of organs, including the lungs. Adiponectin, among the adipocytokines expressed by adipose tissue, is particularly abundant.^1^ Adiponectin acts as a crucial regulator of cytokine responses, participating in numerous physiological processes such as energy metabolism, inflammation, and vascular physiology. In this context, nutritional status emerges as a significant factor in the development and progression of cancer, suggesting its potential involvement in the diagnosis and prognosis of lung cancer.^8^, ^9^, ^10^ In addition, adiponectin is strongly correlated to nutritional status and to body composition. In this scenario, the analyses of body composition are fundamental in nutritional status evaluation both in physiological and pathophysiological conditions. Body composition is a complex of unmodifiable factors such as heredity, sex, aging, and of acquired lifestyle factors which are very modifiable.^11^ In fact, a range of weight‐control strategies, notably exercise, can change the body mass index (BMI).^12^, ^13^ Thus, body composition can be viewed as an aggregate outcome of the cumulative effect of unmodifiable and modifiable factors in the human body. Therefore, body composition indicators may be useful not only as early predictors of lung cancer risk, but also as indicators of lung cancer intervention effectiveness. The interrelationship of body composition and lung cancer is complex and multifactorial, possibly because of differences in ethnicity, nutrition, lifestyle habits, and obesity.^14^ The methods most used to evaluate body composition in clinical practice are based on bicompartmental models and measure, directly or indirectly, fat mass (FM), and free fat mass (FFM). Bioelectrical impedance analysis (BIA) is one of the methodologies that can be used to calculate body composition parameters. BIA is a noninvasive technique that measures human body bioelectrical conductivity value, and it provides an accurate estimate of body composition.^15^, ^16^ Furthermore, the adipose tissue is the organ that is most affected by variations in caloric intake, inflammatory and immune status, influencing nutritional status and body composition. Given this evidence, the aim of this study was to evaluate the variation in body composition and adiponectin levels in lung cancer patients before and after video‐assisted thoracoscopic surgery (VATS) lobectomy.

## METHODS

### Study participants

We performed a retrospective single‐center study which included 30 patients (19 male; 11 female) who underwent VATS lobectomy for lung cancer from September 2021 to May 2022 at the Thoracic Unit of Luigi Vanvitelli University of Naples. The exclusion criteria were patients with cardiovascular diseases, coagulopathies, and respiratory failure, while inclusion criteria were patients with stage 1 or 2 of disease. A control group were also recruited (15 male; 15 female) consisting of age and sex matched volunteered subjects at the Thoracic Unit of Luigi Vanvitelli University of Naples. The control group and lung cancer patients were monitored for anthropometric and biochemical parameters before VATS lobectomy. In addition, lung cancer patients were also monitored after 6 months of surgery. VATS lobectomy is performed using a thoracoscope, which is a thin, flexible tube with a camera and surgical instruments attached to it. Instead of a large incision, VATS lobectomy requires only a few small incisions in the chest. The surgeon inserts the thoracoscope and instruments through these incisions to visualize and operate on the lung.^3^ The surgeon navigates through the chest cavity using the camera's real‐time video feed. The affected lobe is carefully dissected and removed, along with nearby lymph nodes to check for the spread of cancer. Because VATS lobectomy is minimally invasive, patients generally experience less pain, a shorter hospital stay, and a quicker recovery compared with traditional open surgery.^4^, ^5^, ^6^, ^7^ Not all patients are suitable candidates for VATS lobectomy. Factors such as the size and location of the tumor, the stage of cancer, and the patient's overall health play a role in determining the appropriate treatment approach. In addition, two of 30 lung cancer patients are subjected to adjuvant therapy. After surgical intervention, pulmonary rehabilitation consisting of a personalized physical exercise program is also commenced. This study was performed in accordance with the Declaration of Helsinki and approved by the local ethics committee Ethics Committee of University of Campania Luigi Vanvitelli, Naples (approval code: no. 280 on May 16, 2020). Informed consent was signed by both control group and lung cancer patients.

### Anthropometric measurements

Height, weight, and BMI of the lung cancer patients were recorded at baseline before VATs lobectomy and after 6 months of surgery. Bodyweight was measured in a fasting state in the morning with a mechanical balance (±0.1 kg, SECA 700, Hamburg, Germany). BMI was calculated according with the World Health Organization guidelines. As previously reported, body composition was estimated by BIA (Quantum V Segmental Bia, A‐WAVE).

### Biochemical parameters

The samples were collected after an overnight fast (12 h) and stored at −80°C until use. Serum concentration of albumin, C‐reactive protein (CRP), glucose, total cholesterol, high‐density lipoprotein (HDL), and low‐density lipoprotein (LDL) cholesterol and triglycerides were evaluated. In addition, serum concentration of total adiponectin was evaluated by enzyme‐linked immunosorbent assay (ELISA) using a commercial kit (Elabscience).

### Statistical analysis

Statistical analyses were performed using the GraphPad 6 Software, Inc., for Windows, version 6.01. The data are presented as mean (M) ± standard deviation (SD), and statistical significance was set at *p* < 0.05. The Shapiro–Wilk test was used to check the normal distribution of variables. The differences in anthropometric parameters between the cancer and control groups were analyzed with an independent *t*‐test. Data collected from the participants were compared by means of one‐way repeated measure ANOVA, followed by the Tukey's multiple comparisons post hoc test. If the overall F test was significant, Tukey's post hoc comparisons were used. The classification rate was computed by the analysis of the receiver operating characteristic (ROC) curve.^17^

## RESULTS

### Lung cancer patient characteristics

The average age of the patients was 61 years (range: 46–81) with a 95% CI (59.72–64.07). As shown in Table 1, there were 11 (37%) patients with nodules in the right lower lobe (RUL), four (13%) patients with nodules in the left lower lobe (LLL), two (7%) patients with nodules in the middle lobe (ML), seven (23%) patients with nodules in the right lower lobe (RLL), and six (21%) patients with nodules in the left upper lobe (LUL). Nodule diameter (median) was 19 mm and the distance from the visceral pleura was 15 mm. Histology was 16 (53%) patients with adenocarcinoma, two (7%) patients with adenosquamous carcinoma, 11 (37%) patients with squamous carcinoma and one (3%) patient with pleomorphic carcinoma. Surgical resections were 11 patients who underwent right upper lobectomy (37%), two middle lobectomy (7%), seven right lower lobectomy (23%), six left upper lobectomy (20%) and four left lower lobectomy (13%). Histological examination of the lymph nodes showed 13 (43%) patients with metastatic N1 lymph nodes, one (3%) patient with metastatic N1–N2 and one (3%) patient with N2 skip metastasis. The remaining patients had no metastatic lymph nodes.

**TABLE 1:** Principal characteristics of lung cancer patients.

	Patients (*N* = 30)
Side of lung nodules, *n* (%)	
RUL (right upper lobe)	11 (37%)
LLL (left lower lobe)	4 (13%)
ML (middle lobe)	2 (7%)
RLL (right lower lobe)	7 (23%)
LUL (left upper lobe)	6 (21%)
Nodule diameter (mm) median	19
Distance from the visceral pleura (mm) median	15
Histology, *n* (%)	
Adenocarcinoma	16 (53%)
Adenosquamous carcinoma	2 (7%)
Squamous carcinoma	11 (37%)
Pleomorphic carcinoma	1 (6%)
Surgical resections *n* (%)	
Right upper lobectomy	11 (37%)
Middle lobectomy	2 (7%)
Right upper lobectomy	7 (23%)
Left upper lobectomy	6 (20%)
Left lower lobectomy	4 (13%)
Histological examination of the lymph nodes showed	
N1	13 patients (43%)
N1–N2	1 patient (3%)
N2 skip metastasis	1 patient (3%)
N0	15 patients (50%)

### Anthropometric and biochemical futures of lung cancer patients

The anthropometric and biochemical characteristics of patients with lung carcinoma before and after 6 months of VATS lobectomy and the control group are presented in Tables 2 and 3. A reduction in weight, BMI, and body fat mass was observed, with these parameters showing statistically significant decreases. As for the biochemical parameters such as blood glucose and total cholesterol there was not a strong alteration. Furthermore, adiponectin levels in participants statistically increased after lobectomy, while CRP, albumin and triglyceride levels significantly decreased, even when compared with the control group.

**TABLE 2:** Anthropometric and biochemical parameters of lung cancer patients before and after VATS lobectomy and control group.

	Lung cancer patients			
	Before	After		
	VATS lobectomy	VATS lobectomy	Control group	*p*‐value*
Age	61 ± 8.2	‐	61 ± 8.5	ns
Height (m)	1.73 ± 0.03	‐	1.75 ± 0.05	ns
Weight (kg)	80.5 ± 3.33	72.16 ± 3.36	70.1 ± 3.03	0.049
BMI (kg/m^2^)	26.9 ± 4.78	24.8 ± 3.62	22.8 ± 2.91	0.042
Glycemia (mg/dL)	109.33 ± 1.5	110.16 ± 2.4	80.76 ± 1.4	0.67
Total cholesterol (mg/dL)	150.13 ± 10.77	153.91 ± 12.93	130.8 ± 7.30	0.63
HDL (mg/dL)	50.13 ± 1.14	57.76 ± 3.14	62.8 ± 5.92	0.079
LDL (mg/dL)	100.83 ± 6.48	107.57 ± 7.72	68 ± 4.53	0.077
Triglycerides (mg/dL)	115.54 ± 5.27	111.25 ± 6.14	100 ± 5.65	0.029
Total protein (g/dL)	6.85 ± 0.4	5.53 ± 0.4	7.1 ± 3.21	0.55
Albumin (g/dL)	4.05 ± 0.3	3.26 ± 0.7	3.5 ± 0.01	0.016
CRP (mg/mL)	0.89 ± 0.1	0.48 ± 0.07	0.08 ± 0.05	0.007
Adiponectin (μg/mL)	9.81 ± 0.27	10.07 ± 0.7	11.2 ± 0.02	0.022

* The *p*‐value refers to the difference between lung cancer patients before and after VATS lobectomy.

Abbreviations: BMI, body mass index; CRP, C‐reactive protein; HDL, high‐density lipoprotein; LDL; low‐density lipoprotein; VATS, video‐assisted thoracoscopic surgery.

**TABLE 3:** Body composition of lung cancer patients before and after VATS lobectomy.

	Before	After	Control group	*p*‐value*
	VATS lobectomy	VATS lobectomy		
FM (kg)	28.3 ± 1.5	23.9 ± 2.3	26.8 ± 1.5	0.000
FFM (kg)	52.2 ± 1.1	53.3 ± 1.3	51.2 ± 1.4	0.054
BMR (kcal)	1512.4 ± 1.23	1589.9 ± 184	1579.9	0.066
TBW (L)	41.7 ± 1.2	43.3 ± 1.4	44.3 ± 1.2	0.053

* The *p*‐value refers to the difference between lung cancer patients before and after VATS lobectomy.

Abbreviations: BMR, basal metabolic rate; FFM, fat‐free mass; FM, fat mass; TBW, total body water; VATS, video‐assisted thoracoscopic surgery.

### Statistical analysis

#### ANOVA analysis

**FIGURE 1:**
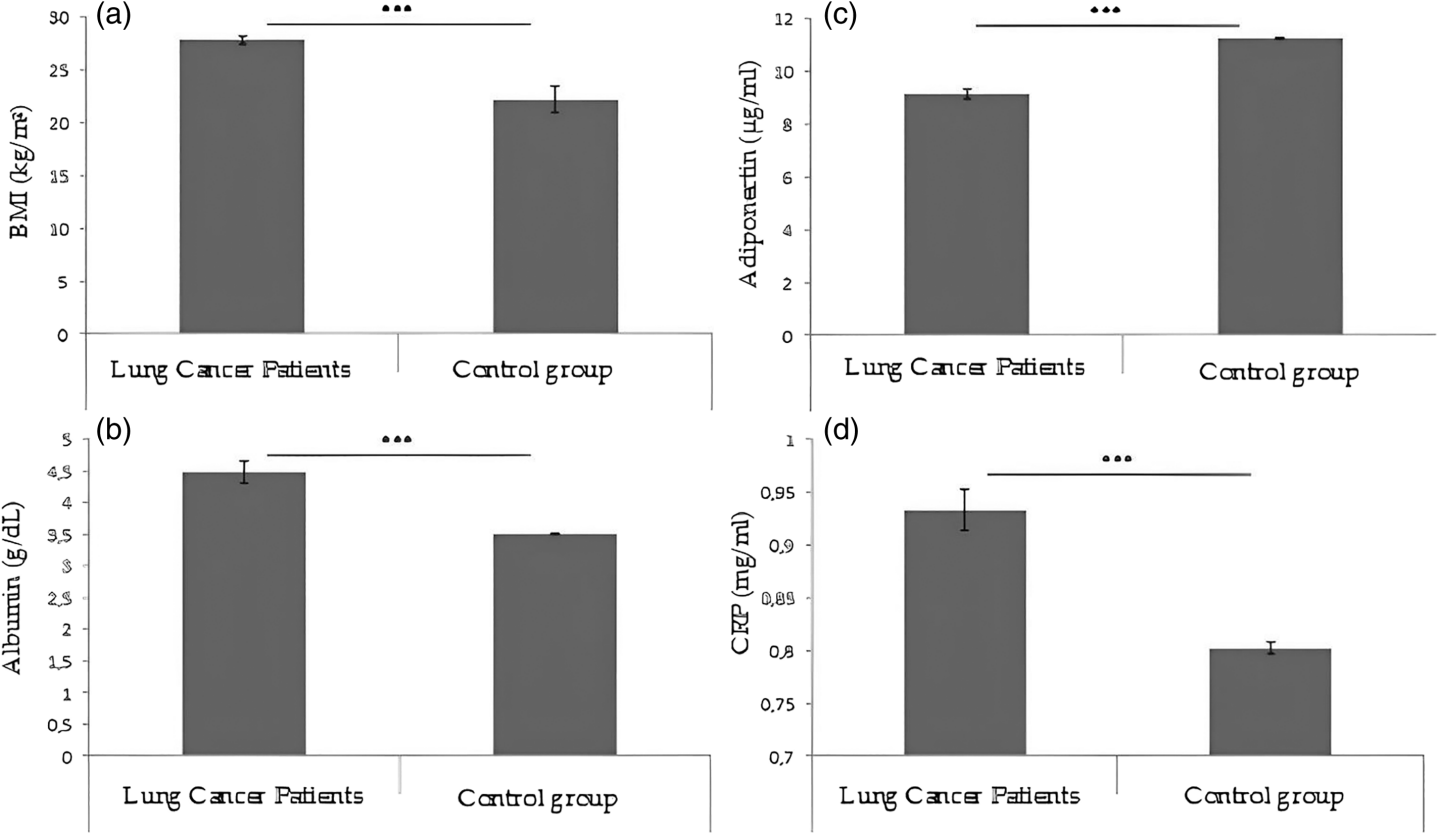
Tukey's multiple comparisons post hoc results. (a) Shows the differences in body mass index (BMI) between lung cancer patients and control group before surgery (*p* < 0.001). (b) Shows the differences in albumin (g/dL) between lung cancer patients and control group before surgery (*p* < 0.001). (c) Shows the differences in adiponectin (μg/mL) between lung cancer patients and control group before surgery (*p* < 0.001). (d) Shows the differences in C‐reactive protein (CRP) (mg/mL) between lung cancer patients and control group before surgery (*p* < 0.001).

The ANOVA showed significant differences between lung cancer patients and the control group (F_(9,290)_ = 14 653; *p* < 0.001) (Figure 1). After surgery there were significant differences between lung cancer patients and control group (F_(9,290)_ = 5118; *p* < 0.001) in the ANOVA; however, the Tukey's multiple comparisons post hoc results showed no significant differences between the cancer and control groups (Figure 1).

#### ROC curve analysis for BMI

**FIGURE 2:**
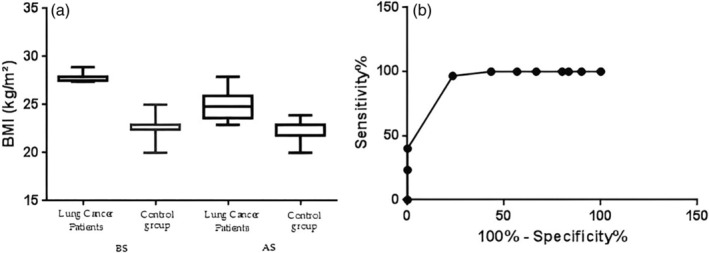
(a) Shows the distribution of body mass index (BMI) data and (b) the receiver operating characteristic (ROC) curve.

**FIGURE 3:**
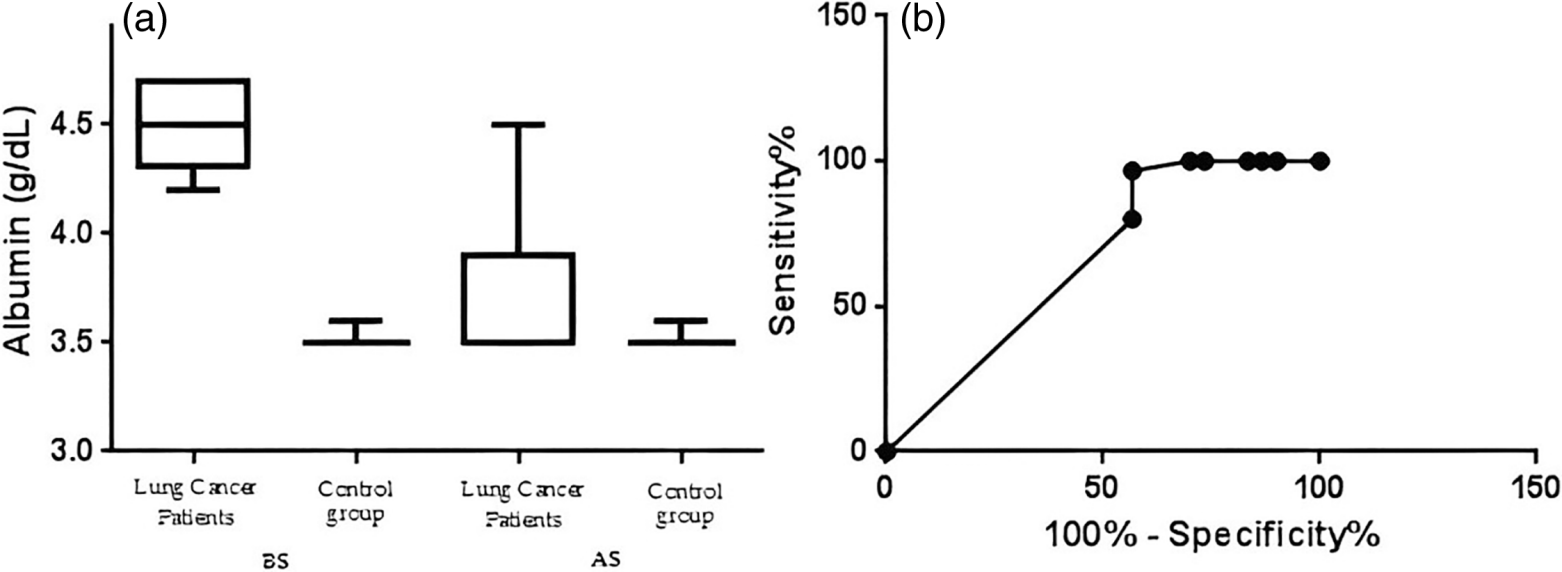
(a) Shows the distribution of albumin (g/dL) data and (b) the receiver operating characteristic (ROC) curve.

**FIGURE 4:**
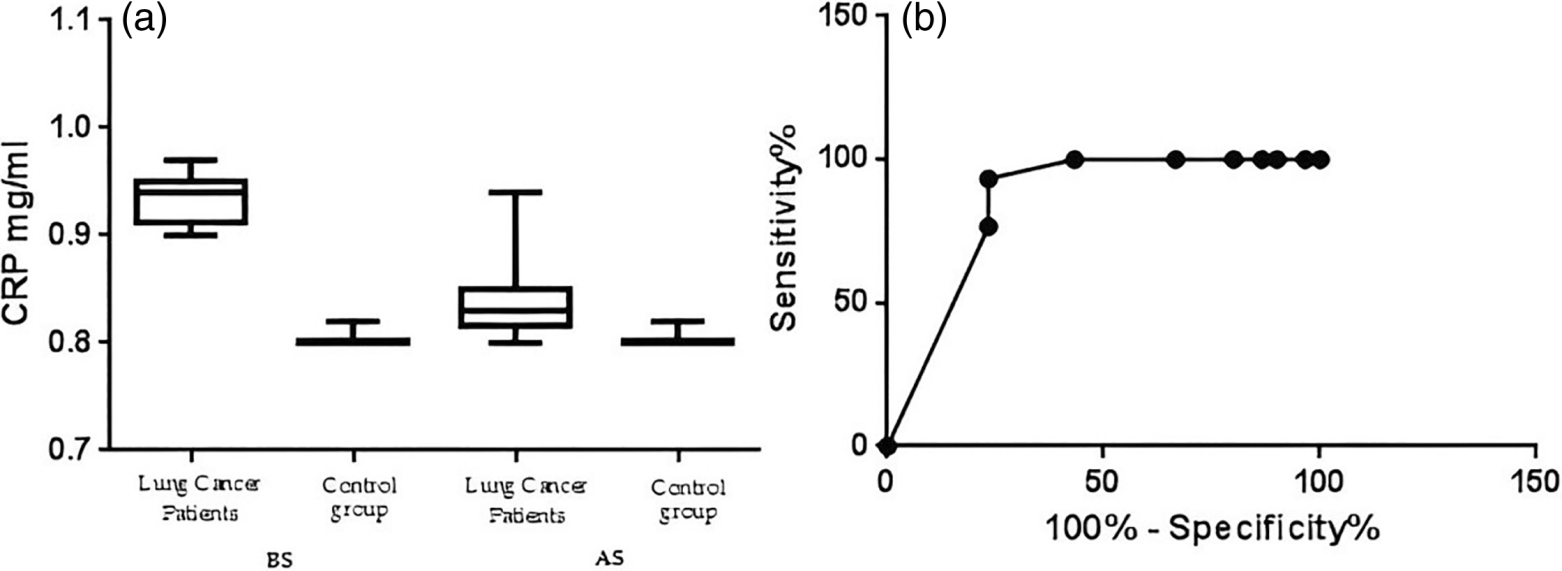
(a) Shows the distribution of C‐reactive protein (CRP) (mg/mL) data and (b) the receiver operating characteristic (ROC) curve.

**FIGURE 5:**
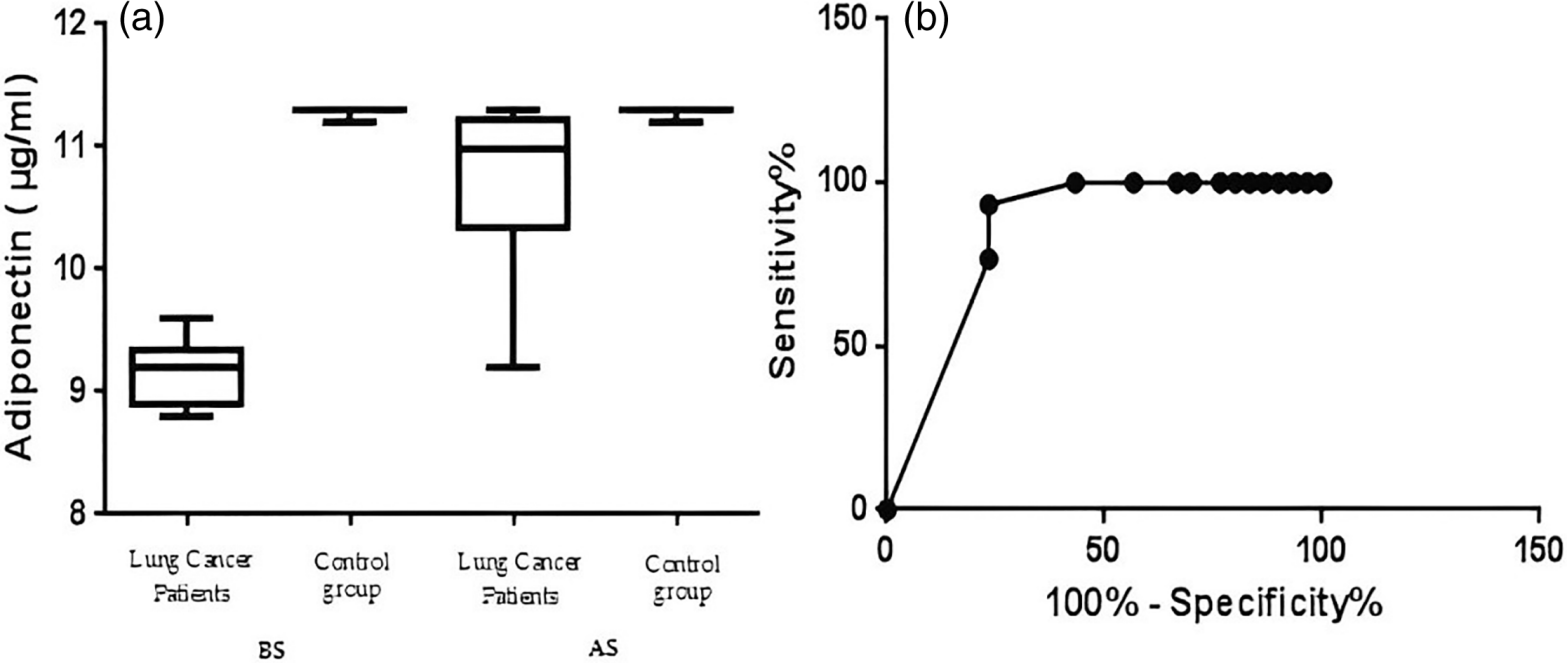
(a) Shows the distribution of adiponectin (μg/mL) data and (b) the receiver operating characteristic (ROC) curve.

In this section, the distribution of the BMI, albumin (g/dL), CRP mg/mL and adiponectin (μg/mL) data before surgery (BS) after surgery (AS), and the ROC curve analysis of sensitivity and specificity are reported. The *p*‐value was from the comparison with the ROC AUC (area under the curve) of specificity versus ROC AUC of sensitivity, *p* < 0.001 (sensitivity 96.67%, specificity 76.67%, area 0.9228 for BMI); *p* < 0.05 (sensitivity 80.0%, specificity 43.33%, area 0.6578 for albumin); *p* < 0.001 (sensitivity 93.33%, specificity 76.67%, area 0.8494 for CRP); and *p* < 0.001 (sensitivity 93.33%, specificity 56.67%, area 0.8391 for adiponectin) as reported in Figures 2, 3, 4, 5.

## DISCUSSION

In this study, we conducted an analysis of body composition through BIA analysis, as well as evaluating the inflammatory status of patients with lung carcinoma before and after VATS lobectomy, comparing them with a control group. Our findings revealed significant alterations in body composition, serum adiponectin levels, and CRP, albumin and triglycerides following surgery. The patients in our study were overweight, and we observed an increase in adiponectin levels after VATS lobectomy compared to before the surgery. In the study we reported a remodulation of body composition and inflammatory status in lung cancer patients after surgery intervention. Interestingly, only two of 30 patients were subjected to adjuvant therapy; for these reasons, the amelioration of body composition is attributable to tumor resection. Body composition plays a significant role in cancer development and disease outcomes.^18^ It can be influenced by factors such as diet, physical activity, genetics, and overall health. Obesity is a key component of body composition, and is associated with an increased risk of developing lung cancer, especially in nonsmokers. Excess body fat, particularly visceral fat, can promote inflammation and the release of inflammatory molecules.^19^, ^20^ This can contribute to the development and progression of lung cancer. Poor body composition, such as a low muscle mass or muscle wasting, can also negatively impact a patient's ability to tolerate cancer treatments and recover from surgery. In addition, body composition and chronic inflammation are factors that influence each other.^21^, ^22^, ^23^ Chronic inflammation can influence body composition by promoting the storage of visceral fat and impairing the body's ability to maintain lean muscle mass. Inflammatory cytokines, such as tumor necrosis factor (TNF) and interleukin‐6 (IL‐6), can disrupt the normal balance of energy metabolism and lead to insulin resistance, which can further promote weight gain and obesity.^24^, ^25^ Furthermore, serum adiponectin levels are inversely correlated to serum cytokine levels, BMI, and body composition.^26^ Adiponectin's involvement in lung cancer provides new insights into the interaction between adipose tissue and the lungs, shedding light on its potential to influence the tumor microenvironment.^27^, ^28^, ^29^ The increase in adiponectin levels after lobectomy is not only due to weight loss but also the elimination of lung cancer.^30^ Adiponectin levels have been strongly correlated with the stage of lung cancer. Moreover, adiponectin plays a role in immune response modulation, reducing B cell lymphopoiesis and T cell responsiveness. Therefore, it actively contributes to the prognosis and outcomes of lung cancer.^31^ Although some studies indicate no significant association between adiponectin levels and lung cancer, many others strongly support that hypoadiponectinemia is a clinical indicator of lung cancer progression. Recent research demonstrated a significant reduction in total adiponectin levels, predominantly affecting high molecular weight adiponectin, in patients with NSCLC compared with healthy subjects.^32^, ^33^ Our patients were overweight before surgery, and we observed changes in body composition and adiponectin levels after surgery. Tumor removal and weight loss may influence adiponectin levels, leading to reduced inflammation, despite lower albumin levels. While the significant role of adiponectin in pulmonary pathophysiological conditions is well‐known, there are conflicting findings regarding adiponectin levels in lung cancer patients. Some studies have not found significant differences in adiponectin concentrations between lung cancer patients and controls, although lower levels have been observed in patients with advanced disease stages.^28^, ^29^ Other studies have reported no significant differences in serum adiponectin levels in patients with advanced NSCLC. Conversely, certain studies have demonstrated a significant increase in serum adiponectin levels in lung cancer patients, although they found no correlation with clinical data, unlike our findings. These inconsistencies may be attributed to various factors, such as age, sex, BMI, and tumor subtypes within the studied population.^30^, ^31^ Another important factor that indicates an amelioration in body composition and inflammation status is the reduction of albumin, CRP, and triglycerides levels, indicating a remodulation of the microenvironment of lung cancer. Inflammation and immune responses play a critical role in shaping the tumor microenvironment.^15^ Chronic inflammation can promote tumor growth and metastasis. The relationships between these biomarkers and the lung cancer microenvironment are complex and multifaceted; elevated CRP levels indicate systemic inflammation, which can create a proinflammatory environment within the tumor microenvironment, potentially promoting cancer cell survival and progression. Low levels of albumin, often seen in lung cancer patients, can be indicative of poor nutritional status and may weaken the immune system, affecting the body's ability to combat the tumor; dysregulation of lipid metabolism, represented by elevated triglycerides, can contribute to chronic inflammation and insulin resistance, which may influence the tumor microenvironment and cancer progression.^16^ In summary, CRP, albumin, and triglycerides are biomarkers that reflect systemic inflammation, nutritional status, and metabolic health. In the context of the tumor microenvironment, these factors may indirectly influence the progression and behavior of lung cancer by shaping the inflammatory and metabolic milieu in which the tumor exists.^18^ In addition, lung cancer patients are subjected to a rehabilitation program, consisting also of physical activity exercise; for these reasons there is a modulation not only in the microenvironment of lung cancer but also systemic modulation.^32^, ^33^ In individuals with lung cancer, physical activity serves as a nonpharmacological approach that can lead to notable improvements. Exercise and physical activity have the capacity to diminish inflammation and activate molecular signaling pathways that facilitate muscle mass development while promoting advantageous metabolic adaptations.^34^ The role of physical activity and exercise extends as a nonpharmacological strategy for lung cancer treatment, offering enhancements in fatigue levels, quality of life, lung function, muscle mass, strength, and psychological well‐being, as evidenced by multiple studies.^35^ From a clinical perspective, the benefits of exercise for lung cancer patients encompass fatigue reduction, enhanced lung function, and improved sleep quality, all contributing to an overall elevation in quality of life. Preoperative exercise for lung cancer patients undergoing surgery diminishes the risk of postoperative lung complications and enhances rehabilitation.^36^ In the case of frail elderly patients with lung cancer undergoing pulmonary resection, engaging in high‐intensity preoperative exercise has been shown to improve rehabilitation outcomes both during hospitalization and in the home environment. This improvement is marked by increased muscle strength and relief from respiratory symptoms. For patients with nonoperable lung cancer, exercise plays a crucial role in preserving lung function and muscle strength.^37^ In addition, the reduction of fat mass contributes to amelioration of inflammatory status, indeed the accumulation of visceral adipose tissue leads to disruptions in the endocrine functionality of adipose tissue, affecting the production of pro‐ and anti‐inflammatory cytokines, confirming the cross talk between lung microenvironment and adipose tissue. A limitation of our study is represented by the small number of patients recruited. Nevertheless, the important results are a stimulus to continue a recruitment and experiment on this cohort, to also understanding the role and molecular mechanism of the “adiponectin system” and body composition in relation to lung cancer establishment.

In conclusion, obesity is often associated with increased infiltration of immune cells into adipose tissue, leading to an imbalance in the production of inflammatory cytokines and adipokines, ultimately promoting tumor development. Various cytokines, such as IL‐1, IL‐6, and TNF‐α, as well as adipokines such as leptin and adiponectin, significantly impact tumor cell proliferation and invasion. Moreover, the risk of obesity‐related cancer development can vary depending on factors such as body composition, fat distribution, and adipose tissue profile. Numerous epidemiological studies have reported a higher risk of developing various types of cancer in individuals with metabolic disorders and low adiponectin expression levels. The findings from our study support the concept that disturbances in adiponectin homeostasis may modulate the processes involved in carcinogenesis. Additionally, considering that physical training and pulmonary rehabilitation can modulate adipokine homeostasis, further exploration of these interventions in lung cancer patient cohorts is warranted. Investigating the intricate interplay between the “adiponectin” system and lung cancer can provide novel insights into the cross‐organ communication that influences tumor growth. Perturbations in the adiponectin system, as observed in patients with lung cancer, can modulate the antiproliferative and anti‐inflammatory effects mediated by adiponectin, thereby offering new therapeutic options for lung cancer patients. Moreover, a comprehensive understanding of body composition, particularly adipose tissue, in cancer development and progression is essential.

## AUTHOR CONTRIBUTIONS

Concept and design: Messina Gaetana, Rita Polito, Alfonso Fiorelli, Fiorenzo Moscatelli, Vincenzo Monda, and Antonietta Monda. Provision of study materials or patients: Giovanni Natale, Carminia M. Della Corte, Carmine Dalia, Paola Bassi, and Beatrice Leonardi. Collection and assembly data: Antonella De Maria, Antonietta Monda, Rita Polito, Gabriella Marsala and Maria A. Puca. Data analysis and interpretation: Gabriella Marsala, Marcellino Monda and Giuseppe Vicario. Manuscript writing: All authors. Final approval of manuscript: All authors.

## FUNDING INFORMATION

This research received no external funding.

## CONFLICT OF INTEREST STATEMENT

The authors declare no conflict of interest.
